# Special Issue on Marine Toxins

**DOI:** 10.3390/md7010019

**Published:** 2009-01-29

**Authors:** Alejandro M.S. Mayer

**Affiliations:** Department of Pharmacology, Chicago College of Osteopathic Medicine, Midwestern University, 555 31st Street, Downers Grove, Illinois 60515, USA; Tel. +1-630 515 6951; Fax +1-630 515 6295; E-mail: amayer@midwestern.edu

The special issue on Marine Toxins of the Open Access journal *Marine Drugs* (ISSN 1660-3397, http://www.mdpi.com/journal/marinedrugs/) presents twenty four contributions which were received from distinguished investigators currently working in Canada, China, France, Germany, Iran, Italy, Japan, Portugal, Russian Federation, Slovenia, South Africa, Spain, and the United States. The reviews and research articles provide the interested reader with a global view of marine toxins research during 2007–2008.

## Review Articles

Fourteen review articles on several marine toxins and their impact on animal and human health are presented. Leone and colleagues from Italy and the Russian Federation [1] provide an update on the molecular structure of **endotoxins** from marine Gram-negative bacteria, and discuss their potential as lead candidates in the development of novel drugs to prevent septic shock. Turk and colleagues from Slovenia [2] present the current status of the mechanisms of toxicity of **3-alkylpyridinium polymers** isolated from the marine sponge *Reniera sarai.* Twiner and colleagues from Charleston, South Carolina [3] review recent advances on the chemistry of various **azaspiracid** analogs, the putative progenitor algal species, as well as *in vitro* and *in vivo* data on the toxicology to human health. Paz and colleagues from Spain [4] discuss in their review the origin of **yessotoxins**, marine polyether toxins isolated from the scallop *Patinopecten yessoensis*, the producer organisms and vectors, the chemical structures, the biosynthetic origin and toxicological properties, and the potential risks of yessotoxins to human health. Berry and colleagues from Miami, Florida [5] review freshwater and marine **cyanobacterial toxins** as allelochemicals with potential applications as algaecides, herbicides and insecticides, including recent results from investigations into cyanobacterial toxins from the Florida Everglades and associated waterways. Klisch and Häder from Germany [6] provide a comprehensive review on UV-protective **mycosporine-like amino acids** which are produced by freshwater and marine cyanobacteria, dinoflagellates and diatoms, highlighting both the common characteristics of  these chemicals with marine toxins, as well as their differences. Pulido from Canada [7] contributes a systematic and comprehensive review of amnesic shellfish poisoning, a brain pathology induced by **domoic acid**, a marine toxin produced by red alga *Chondria armata* and planktonic diatoms of the genus *Pseudo-nitzschia,* and currently considered a risk to global health of humans and wildlife. Noguchi and Arakawa from Japan [8] systematically review the distribution and accumulation of **tetrodotoxin,** a potent neurotoxin produced in marine bacteria, which has been shown to accumulate both in pufferfish as well as other marine organisms, and has become a significant human health problem. Ramsdell and Zabka from Charleston, South Carolina and Sausalito, California, respectively, review [9] the potential prenatal toxicity and exposure susceptibility to **domoic acid** in Californian sea lions, with the purpose of providing a useful tool to forecast prenatal toxicity. Deeds and colleagues from South Africa and the USA [10] review the importance of filter feeding (traditional) and non-filter feeding (non-traditional) vectors for **saxitoxin** making special reference to paralytic shellfish poisoning in humans. Wang from the People’s Republic of China [11] contributes a review on a group of neurotoxins produced by marine dinoflagellates, organisms responsible for harmful algal blooms, and which have been shown to cause paralytic shellfish poisoning, neurotoxic shellfish poisoning, ciguatera fish poisoning, and azaspiracid poisoning. Watkins and colleagues from Tallahassee and Miami, Florida [12] review the known epidemiology of **brevetoxins**, neurotoxins primarily produced by the dinoflagellate *Karenia brevis,* as well as recommendations for improved neurotoxic shellfish poisoning prevention associated with the consumption of molluscan shellfish contaminated with brevetoxins. Friedman and colleagues from Alabama, Florida, Georgia, Hawaii, and South Carolina [13] present a comprehensive and systematic review of the treatment, prevention and management of **ciguatera fish poisoning**, a global seafood-toxin illness that causes significant gastrointestinal, neurologic and/or cardiovascular symptoms. Mariottini and colleagues from Italy [14] contribute to toxicological research on **Cnidarian venoms** with their review of the distribution, ecology, toxicity, and epidemiology of *Pelagia noctiluca* (Forsskål, 1775), the most venomous Mediterranean jellyfish.

## Research Articles

Ten research articles provide recent experimental results with a number of marine toxins. Mayer and colleagues from Downers Grove, Illinois [15] challenge the **domoic acid**-microglia direct activation hypothesis. Their experimental observations demonstrate that a short term (4 to 24 hours) *in vitro* exposure to **domoic acid**, the causative agent of amnesic shellfish poisoning in humans, does not appear to activate rat neonatal brain microglia, and the concomitant release of the neuroinflammatory mediators. Cheng and colleagues from Florida, Georgia and New Mexico [16], evaluate sampling and analysis techniques for **microcystin** aerosols, and show that these cyclic peptide cyanotoxins are transferred from water to air via a bubble-bursting process, a finding of considerable significance in view of the fact that microcystin inhalation is a likely route of human exposure. Amzil and colleagues from France [17], report the first detection of **spirolide-A** in shellfish from the Atlantic coast (Southern Brittany), and **pectenotoxin-2**, on the Mediterranean coast (the island of Corsica), findings that may provide a scientific basis for establishing regulatory control. Banack and colleagues from California and Wyoming [18], report the production of the neurotoxic non-protein **amino acid β-methylamino-L-alanine** in laboratory cultures of a free-living marine *Nostoc* species employing five  completely different methods, a finding that may contribute to ongoing studies on the environmental bioaccumulation of this toxin, which has been associated with amyotrophic lateral sclerosis and parkinsonism in the island of Guam. Fleming and colleagues from Florida, Georgia and Ohio [19] demonstrate that an automated call processing menu system that allows callers to access bilingual **harmful algal blooms** information provides useful information for the majority of callers, and decreases the routine informational call workload for Poison Information Specialists. Gill and colleagues from Ottawa, Canada and Tehran, Iran [20] report that **domoic acid** activation of hippocampus and brain stem astrocytes results in expression of early response gene c-jun, the glutamate receptor GluR 2, as well as chemokine, cytokine and apoptotic genes, thus suggesting that astrocytes may become a potential target for pharmacological interventions designed to treat amnesic shellfish poisoning. Lefebvre and colleagues from Seattle, Washington and Lisbon, Portugal [21] characterize the intracellular and extracellular **saxitoxin** levels in both field and cultured *Alexandrium* spp. samples from Sequim Bay, Washington, demonstrating that gonyautoxin 1 and 4 constitute 65 ± 9.7 % of the total paralytic shellfish poisoning toxins present. Perez and colleagues from Florida [22] assess both **okadaic acid** producing and non-producing species of the genus *Prorocentrum* for the presence of polyketide synthase (PKS) encoding genes. Their data, which results from PKS phylogenetic analysis, indicates that a *Prorocentrum*-specific bacterial PKS clade appears to exist, along with more dispersed *Prorocentrum*-derived sequences, suggesting that okadaic acid production may derive from one of the bacterial sources investigated. Walsh and colleagues from Florida [23] report on the immunotoxic effects of **brevetoxin**, a toxin produced by the dinoflagellate, *Karenia brevis.* Studies on apoptosis and cellular metabolism in a leukemic T cell line (Jurkat*) in vitro* demonstrate that brevetoxin congeners vary in their respective effects on Jurkat cells, with PbTx-2 and PbTx-6 eliciting greater cellular effects as compared to PbTx-3. Backer and colleagues from Florida, Georgia, Oregon and New Mexico [24] report the first study to document that water-based recreational activities in a small lake, where a *Microcystis aeruginosa* bloom has emerged, may expose people to very low concentrations of aerosol-borne **microcystins.**

The 24 reviews and research articles which are part of this special issue of Marine Toxins, highlight both potential risks associated with marine toxins, as well as potential uses of these chemically diverse marine chemicals. Thus by highlighting current research in marine toxins from a global perspective, the Guest Editor hopes that this special issue of Marine Toxins will contribute in a concrete and perhaps significant way to the ongoing exploration of the ocean’s chemical [25,26] and toxin biodiversity by scientists worldwide.
